# Neonatal BCG Vaccination Reduces Interferon-γ Responsiveness to Heterologous Pathogens in Infants From a Randomized Controlled Trial

**DOI:** 10.1093/infdis/jiaa030

**Published:** 2020-01-28

**Authors:** Bridget Freyne, Nicole L Messina, Susan Donath, Susie Germano, Rhian Bonnici, Kaya Gardiner, Dan Casalaz, Roy M Robins-Browne, Mihai G Netea, Katie L Flanagan, Toby Kollmann, Nigel Curtis, Melbourne Infant Study: BCG for Allergy and Infection Reduction (MIS BAIR) Group, Veronica Abruzzo, Katie Allen, Clare Morrison, Anne-Louise Ponsonby, Peter Vuillermin

**Affiliations:** 1 Infectious Diseases and Microbiology Group, Murdoch Children’s Research Institute, Royal Children’s Hospital Melbourne, Parkville, Australia; 2 Department of Paediatrics, The University of Melbourne, Parkville, Australia; 3 Institute of Infection and Global Health, The University of Liverpool and The Malawi-Liverpool Wellcome Trust Research Programme, Blantyre, Malawi; 4 Clinical Epidemiology and Biostatistics Unit, Murdoch Children’s Research Institute, Parkville, Australia; 5 Department of Paediatrics, Mercy Hospital for Women, Heidelberg, Australia; 6 Department of Microbiology and Immunology, The University of Melbourne, Parkville, Australia; 7 Department of Internal Medicine, Radboud Institute for Molecular Life Sciences, Radboud University Nijmegen Medical Center, Nijmegen, The Netherlands; 8 Radboud Center for Infectious Diseases, Radboud University Nijmegen Medical Center, Nijmegen, The Netherlands; 9 University of Tasmania, Launceston, Australia; 10 Monash University, Clayton, Australia; 11 Department of Experimental Medicine, University of British Columbia, Vancouver, Canada; 12 Department of Pediatrics, University of British Columbia, Vancouver, Canada

**Keywords:** BCG, immunization, heterologous, nonspecific effects, innate immunity, infants

## Abstract

**Background:**

BCG vaccination has beneficial nonspecific (heterologous) effects that protect against nonmycobacterial infections. We have previously reported that BCG vaccination at birth alters in vitro cytokine responses to heterologous stimulants in the neonatal period. This study investigated heterologous responses in 167 infants in the same trial 7 months after randomization.

**Methods:**

A whole-blood assay was used to interrogate in vitro cytokine responses to heterologous stimulants (killed pathogens) and Toll-like receptor (TLR) ligands.

**Results:**

Compared to BCG-naive infants, BCG-vaccinated infants had increased production of interferon gamma (IFN-γ) and monokine induced by gamma interferon (MIG) (CXCL9) in response to mycobacterial stimulation and decreased production of IFN-γ in response to heterologous stimulation and TLR ligands. Reduced IFN-γ responses were attributable to a decrease in the proportion of infants who mounted a detectable IFN-γ response. BCG-vaccinated infants also had increased production of MIG (CXCL9) and interleukin-8 (IL-8), and decreased production of IL-10, macrophage inflammatory protein-1α (MIP-1α), and MIP-1β, the pattern of which varied by stimulant. IL-1Ra responses following TLR1/2 (Pam3CYSK4) stimulation were increased in BCG-vaccinated infants. Both sex and maternal BCG vaccination status influenced the effect of neonatal BCG vaccination.

**Conclusions:**

BCG vaccination leads to changes in IFN-γ responsiveness to heterologous stimulation. BCG-induced changes in other cytokine responses to heterologous stimulation vary by pathogen.

## Teaser

Neonatal BCG vaccination results in a decreased proportion of infants mounting an IFN-γ response to heterologous stimulation at 7 months of age. Both sex and maternal BCG vaccination status influenced the effect of BCG vaccination on heterologous cytokine responses.

BCG vaccination at birth is associated with decreased mortality in infants [1, 2]. This is proposed to be due to heterologous (“off-target” or “non-specific”) protection against early-life infections, an effect thought to result from the immunomodulatory properties of BCG [3, 4]. The beneficial nonspecific effects of BCG vaccine persist beyond the neonatal period. BCG vaccination is associated with reduced incidence of lower respiratory tract infections in childhood and sepsis in the first year of life [5, 6] and protection against eczema in infants with an atopic disposition [7]. Maternal BCG, delivery method, and gestational age have been reported to modify the nonspecific effects of BCG [8, 9].

The mechanisms underlying the beneficial heterologous effects of BCG vaccination remain unclear [10]. We have previously shown that in vitro cytokine responses to heterologous stimulants differ between BCG-vaccinated and BCG-naive neonates [11]. The longer-term heterologous immunological effects induced by neonatal BCG are poorly defined but are likely to be different. Maturational changes mean the immune response in infancy is different to that of neonates [12–14]. In addition, a central hypothesis in the literature on the nonspecific effects of vaccination is that live vaccines confer protection against all-cause mortality, which is negated by subsequent nonlive vaccines in the first 6 months of life [15]. In the current study, we report the effect of neonatal BCG vaccination on heterologous cytokine responses in infants from a randomized controlled trial at 7 months of age.

## METHODS

**Figure 1.**
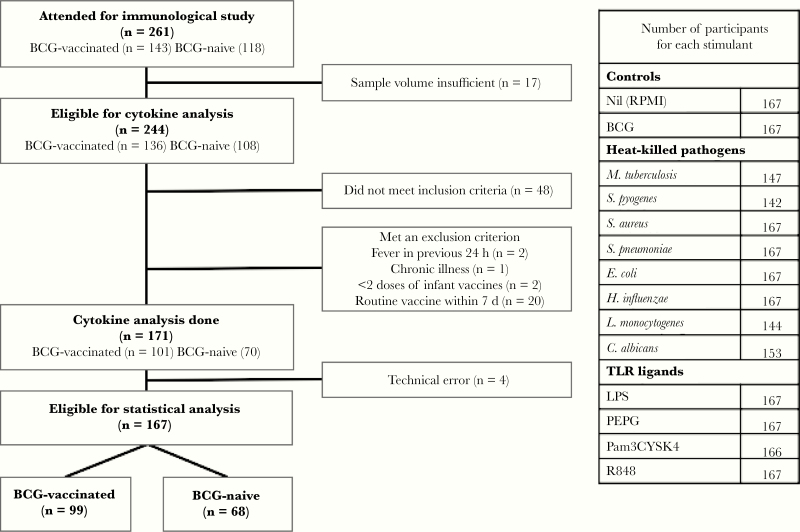
Flow diagram showing participant inclusion. Table shows number of individual stimulations done for each stimulant. Abbreviations: LPS, lipopolysaccharide; PEPG, peptidoglycan; TLR, Toll-like receptor.

Participants were a subset of infants recruited from the Melbourne Infant Study: BCG for Allergy and Infection Reduction (MIS BAIR), in which neonates were randomized to vaccination with BCG-Denmark 0.05 mL intradermally or no BCG vaccination (ClinicalTrials.gov identifier NCT01906853). The inclusion and exclusion criteria for MIS BAIR are described elsewhere [16]. MIS BAIR participants were invited to attend a 7-month study visit to provide a blood sample. All participants with a blood sample from December 2014 to April 2016 were recruited to this substudy. Inclusion criteria for cytokine analysis were as follows: (1) sufficient blood for all stimulants, or (2) participant provided sample in previous study [11]. Exclusion criteria were fever in the previous 24 hours, chronic illness, < 2 doses of the routine scheduled vaccines, any vaccination within the last 7 days, and any blood products since birth (Figure 1).

Venous blood was collected and in vitro stimulation assays were done as described previously [11]. In brief, whole blood, diluted 1:1 with RPMI 1640 medium (GlutaMAX Supplement, HEPES, Gibco, Life Technologies), was stimulated at 37°C (5% carbon dioxide:air) for 20 (± 2) hours with RPMI alone (unstimulated control), BCG-Denmark (Serum Statens Institut, Denmark) 75 μg/mL, killed *Mycobacterium tuberculosis* 1.0 × 10^6^ colony-forming units (CFU)/mL, *Escherichia coli* 1.0 × 10^6^ CFU/mL, *Haemophilus influenzae* type B 1.0 × 10^6^ CFU/mL, *Staphylococcus aureus* 1.0 × 10^7^ CFU/mL, *Streptococcus pyogenes* 1.0 × 10^7^ CFU/mL, *Streptococcus pneumoniae* serotype 15C (nonvaccine serotype) 1.0 × 10^7^ CFU/mL, *Listeria monocytogenes* 1.0 × 10^7^ CFU/mL, *Candida albicans* 1.0 × 10^6^ CFU/mL, and the Toll-like receptor (TLR) ligands lipopolysaccharide (LPS; TLR4 agonist) 100 ng/mL, resiquimod (R848; TLR 7/8) 3.5 μg/mL, peptidoglycan (PEPG; TLR 2/4) 10 μg/mL, and (S)-(2,3-bis(palmitoyloxy)-(2-RS)-propyl)-*N*-palmitoyl-(R)-Cys-(S)-Ser-(S)-Lys4OH,trihydrochloride (Pam3CSK4; TLR 1/2) 1 μg/mL (all from InvivoGen). When insufficient blood was available for all stimulations, a predetermined priority order was used. The following cytokines were measured in supernatants using an xMAP Luminex 200 Analyser as described previously: interferon gamma (IFN-γ), tumor necrosis factor alpha (TNF-α), interleukin-6 (IL-6), IL-1β, macrophage migration inhibitory factor (MIF), IL-8 (CXCL8), monokine induced by gamma interferon (MIG) (CXCL9), interferon-gamma induced protein (IP-10) (CXCL10), monocyte chemoattractant protein-1 (MCP-1) (CCL2), macrophage inflammatory protein-1α (MIP-1α) (CCL3), MIP-1β (CCL4), IL-10, and IL-1Ra [11]. Study staff were blinded to the BCG vaccination status of participants.

Statistical analysis was done using Stata version 13.1 software. Cytokine results below the lower limit of detection were assigned a value of half the lowest detectable value. There were 14 values above the upper limit of detection; these were excluded from the analysis. Proportions of detectable values for each cytokine are shown in Supplementary Table 1.

Prior to analysis, all cytokine data were log-transformed. Regression analyses, with the log-transformed value of the unstimulated cytokine values as a covariate, was used to determine the effect of BCG vaccination, maternal BCG vaccination status, and infants’ sex on cytokine production in response to each stimulant. This approach was used in all regression analyses to account for variability in unstimulated samples and avoids the need for nil subtraction. For cytokine/stimulant pairs with normally distributed data (Supplementary Figure 1), linear regression was used and results are presented as geometric mean ratio (GMR) with 95% confidence interval (CI). For cytokine/stimulant pairs with nonnormally distributed data (Supplementary Figure 1), quantile regression was used and results are presented as differences in the medians with 95% CI.

Subgroup analysis (sex and maternal BCG vaccination status) was done when there was statistical evidence of an interaction between BCG vaccination and the subgroup variable, as determined by interaction analysis (Supplementary Table 4). Given the smaller numbers of participants in subgroup analyses, quantile regression was used, with bootstrapping as required.

Multivariable analysis was done to assess the effect of sex, delivery method, age at infant BCG vaccination (< 48 or ≥ 48 hours of life), maternal BCG vaccination status, age at blood sample, and the number of doses of routine vaccinations received. Multivariable analyses, with the unstimulated values as a covariate, were done using linear or quantile regression as per the corresponding cytokine/stimulant pair in the primary univariate analysis.

For cytokines that showed a dichotomous response, participants were classified according to whether cytokine concentrations in the stimulated supernatants were above (“responders”) or below (“nonresponders”) the lower limit of detection. Fisher exact test was used to compare the proportion of “responders” between BCG-vaccinated and BCG-naive participants. To assess the contribution of this observation to the results of the primary analysis, a sensitivity analyses was done for the effect of BCG vaccination when “nonresponders” were excluded.

The study was approved by the research ethics committees of the Royal Children’s Hospital (HREC 33025) and the Mercy Hospital for Women (HREC R12/28).

## RESULTS

Demographic features of participants were similar between BCG-vaccinated and BCG-naive participants (Table 1). In unstimulated samples, BCG-vaccinated infants had lower overall cytokine concentrations (particularly IL-10, MIF, IP-10, and IL-8) compared with BCG-naive infants (Table 2). In response to all stimulants, there was marked interindividual variability in cytokine production (Supplementary Figure 1). For IFN-γ, IP-10, IL-1Ra, and IL-1β, there was evidence of dichotomous responses, that is, participants who did or did not mount a measurable cytokine response following stimulation.

**Table 1.** Demographic Characteristics of the Study Participants

Characteristic	BCG-vaccinated	BCG-naive	Total
All participants	99 (59.3)	68 (40.7)	167 (100)
Sex			
Male	57 (57.6)	37 (54.4)	94 (56.3)
Female	42 (42.4)	31 (45.6)	73 (43.7)
Race/ethnicity			
White	79 (79.8)	57 (83.8)	136 (81.4)
Asian	4 (4.0)	3 (4.4)	7 (4.3)
Mixed ethnicity	4 (4.0)	2 (3.0)	6 (3.6)
Middle Eastern	1 (1.0)	0 (0.0)	1 (0.5)
Missing	11 (11.2)	6 (8.8)	17 (10.2)
Mode of delivery			
Vaginal	63 (63.6)	40 (58.8)	103 (61.7)
Cesarean	36 (36.4)	28 (41.2)	64 (38.3)
Maternal BCG vaccination			
Yes	21 (21.2)	12 (17.7)	33 (19.8)
No	73 (73.8)	51 (75.0)	124 (74.2)
Unknown	5 (5.0)	5 (7.3)	10 (6.0)
Maternal education level			
Completed high school	15 (15.2)	8 (11.8)	23 (13.8)
Trade certificate	12 (12.1)	8 (11.7)	20 (12.0)
University degree	48 (48.5)	31 (45.6)	79 (47.3)
University higher degree	21 (21.2)	18 (26.5)	39 (23.4)
Other	3 (3.0)	3 (4.4)	6 (3.6)
No. of other children < 5 y of age in house			
0	54 (54.6)	45 (66.2)	99 (59.3)
1	36 (36.4)	20 (29.4)	56 (33.5)
2	9 (9.1)	3 (4.4)	12 (7.2)
Participant attends childcare			
Yes	7 (7.1)	5 (7.4)	12 (7.2)
No	92 (92.9)	63 (92.6)	155 (92.8)
Day of life of BCG vaccination			
Mean (SD)	2.0 (1.6)	…	…
Median (IQR)	1 (0–6)	…	…
Age at 7-mo blood sample, d			
Mean (SD)	201 (23.1)	200 (22.9)	201 (23.0)
Median (IQR)	197 (184–223)	200 (182–218)	199 (182–221)
Interval between BCG vaccination and 7m blood sample, d			
Mean (SD)	199.3 (23.2)	…	…
Median (IQR)	196 (161–238)	…	…
No. of routine immunization^a^ doses prior to 7m blood sample			
2	51 (51.5)	34 (50.0)	85 (50.9)
3	47 (47.5)	33 (48.5)	80 (47.9)
Not recorded	1 (1.0)	1 (1.5)	2 (1.2)
BCG vaccine batch			
1	5 (5.1)	…	…
2	85 (85.9)	…	…
3	9 (9.1)	…	…

Data are shown as No. (%) unless otherwise indicated.

Abbreviations: IQR, interquartile range; SD, standard deviation.

^a^Routine immunizations: Infanrix Hexa (hepatitis B, diphtheria-tetanus-pertussis, *Haemophilus influenzae* type B, inactivated poliovirus); Prevenar 13 (13-valent pneumococcal conjugate vaccine); rotavirus (Rotarix or RotaTeq).

**Table 2.** Univariate Analysis for the Effect of Neonatal BCG Vaccination Versus No BCG Vaccination on Unstimulated Cytokine Levels

Cytokine	GMR (95% CI)	*P*-Value
IL-10	0.59 (0.43–0.81)	.001
TNF-α	0.80 (0.60–1.08)	.14
IL-6	0.90 (0.65–1.25)	.53
MIF	0.78 (0.62–0.99)	.04
MIG	0.79 (0.60–1.08)	.14
IP-10	0.64 (0.49–0.86)	.003
IL-8	0.61 (0.41–0.93)	.02
MIP-1α	0.65 (0.41–1.03)	.06
MIP-1β	0.81 (0.57–1.17)	.26
IL-1Ra	0.87 (0.52–1.46)	.59
Cytokine	GMR (95% CI)	*P*-Value
	Difference in Median (95% CI)	
IFN-γ	1.39 × 10^-17^ (–1.57–1.57)	1
IL-1β	6.94 × 10^-18^ (–5.14–5.14)	1
MCP-1	562.05 (–675.46 to 1799.56)	.37

Results are shown as GMR for normally distributed data and difference in median from nonparametric quantile regression for data that were not normally distributed.

Abbreviations: CI, confidence interval; GMR, geometric mean ratio; IFN, interferon; IL, interleukin; IP, interferon-gamma induced protein; MCP, monocyte chemoattractant protein; MIF, macrophage migration inhibitory factor; MIG, monokine induced by gamma interferon; MIP, macrophage inflammatory protein; TNF, tumor necrosis factor.

**Figure 2.**
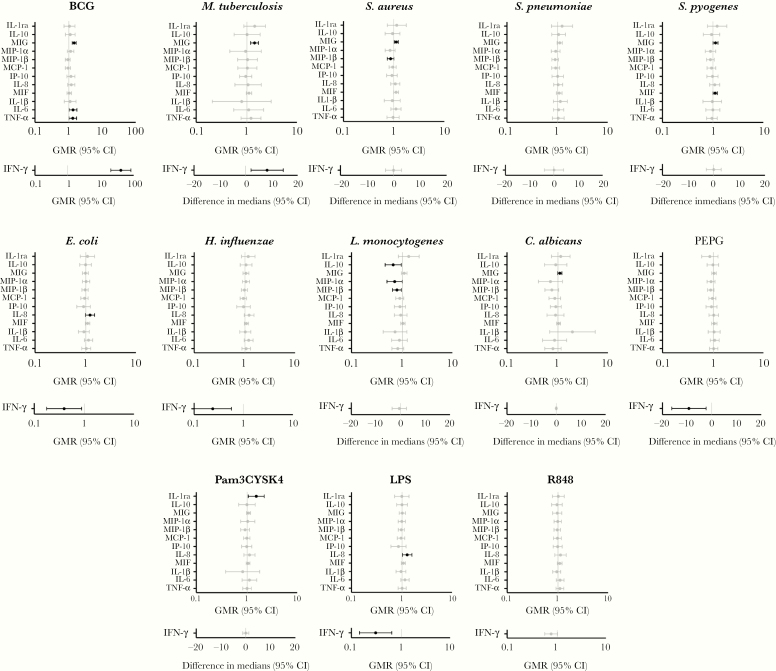
The effect of neonatal BCG vaccination vs no BCG vaccination on cytokine responses to heterologous stimulants. Significant results *P* < .05 are depicted in black. Geometric mean ratio > 1.0 indicates that cytokine levels were higher in BCG-vaccinated infants compared with BCG-naive infants. Data that were not normally distributed were analyzed using quantile regression and differences in medians (95% confidence intervals). As this only applied to interferon gamma (IFN-γ), to aid interpretation, all IFN-γ analyses are displayed on stand-alone axes irrespective of the type of analysis done. Abbreviations: CI, confidence interval; GMR, geometric mean ratio; IFN, interferon; IL, interleukin; IP, interferon-gamma induced protein; LPS, lipopolysaccharide; MCP, monocyte chemoattractant protein; MIF, macrophage migration inhibitory factor; MIG, monokine induced by gamma interferon; MIP, macrophage inflammatory protein; PEPG, peptidoglycan; TNF, tumor necrosis factor.

In response to the specific mycobacterial antigens BCG and *M. tuberculosis*, BCG-vaccinated infants had higher production of IFN-γ and MIG compared with BCG-naive infants, and also higher TNF-α and IL-6 following stimulation with BCG (Figure 2 and Supplementary Table 1). In response to heterologous stimulation with the Gram-positive bacteria *S. pyogenes* and *S. aureus*, BCG-vaccinated infants had differential production of chemokines including MIF, MIG, and MIP-1β. Chemokine production in response to stimulation with the intracellular pathogens (*L. monocytogenes* and *C. albicans*) was also altered in BCG-vaccinated infants (Figure 2 and Supplementary Table 1). In response to stimulation with the Gram-negative bacteria *E. coli* and *H. influenzae*, and the TLR agonist LPS, BCG-vaccinated infants had decreased IFN-γ responses and increased IL-8 response compared with BCG-naive infants. In response to stimulation with TLR agonists Pam3CYSK4 (TLR1/2) and PEPG (TLR2), BCG-vaccinated infants had increased IL-1Ra production and decreased IFN-γ production, respectively (Figure 2 and Supplementary Table 1).

**Figure 3.**
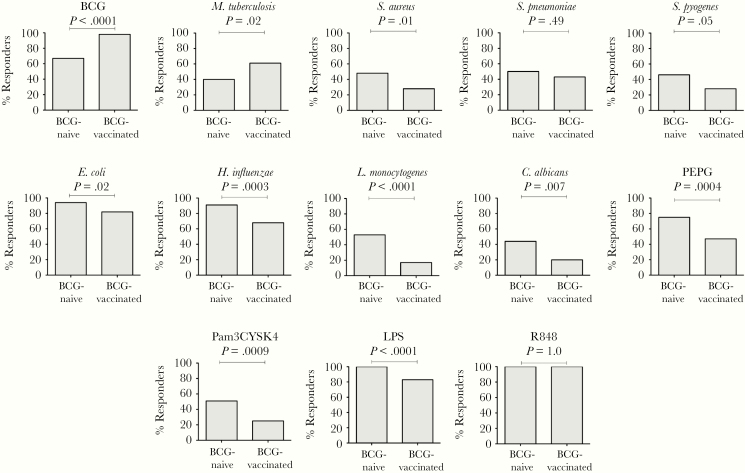
Proportion of BCG-vaccinated and BCG-naive participants with production of interferon gamma (IFN-γ) in response to heterologous stimulation (“responders”) (Fisher exact test). Data for R848 are not included as IFN-γ responses were above the lower limit of detection for all participants.

There was a dichotomous IFN-γ response to all stimulants aside from R848, with a distinct separation between “responders” and “nonresponders.” For the specific mycobacterial antigens, BCG and *M. tuberculosis*, there was a higher proportion of IFN-γ responders in the BCG-vaccinated group compared to the BCG-naive group (Figure 3 and Supplementary Table 2). Conversely, for the heterologous stimulants, the proportion of IFN-γ responders was significantly lower in BCG-vaccinated infants for all stimulants with the exception of *S. pneumoniae* (Figure 3 and Supplementary Table 2). IP-10, IL-1Ra, and IL-1β responses showed a similar pattern; however, differences in the proportion of responders were not statistically significant between BCG-vaccinated and BCG-naive infants (Supplementary Table 2).

A sensitivity analysis done after removal of all nonresponders showed no significant difference in IFN-γ responses to heterologous stimulants between BCG-vaccinated and BCG-naive infants, revealing that the observed difference in IFN-γ GMRs was attributable to the change in proportion of “responders”(Supplementary Table 3). In contrast, the difference in the specific IFN-γ response to in vitro BCG stimulation between BCG-vaccinated and BCG-naive infants was still observed.

Infants’ sex also independently influenced cytokine responses. Compared with girls, boys had increased production of both MCP-1 and MIP-1β in response to almost all stimulants (Supplementary Table 1 and Supplementary Figure 2). Boys also had increased production of IFN-γ in response to stimulation with BCG, *E. coli*, *H. influenzae*, and PEPG, and TNF-α in response to most stimulants, particularly *E. coli*, *H. influenzae*, LPS, and R848 (Supplementary Table 1 and Supplementary Figure 2). MIG production was also higher in boys following stimulation with *E. coli*. Conversely, boys had decreased production of IL-1Ra in response to *C. albicans* and IL-1β in response to *L. monocytogenes*, and in response to Pam3CYSK4 (Supplementary Table 1 and Supplementary Figure 2).

**Figure 4.**
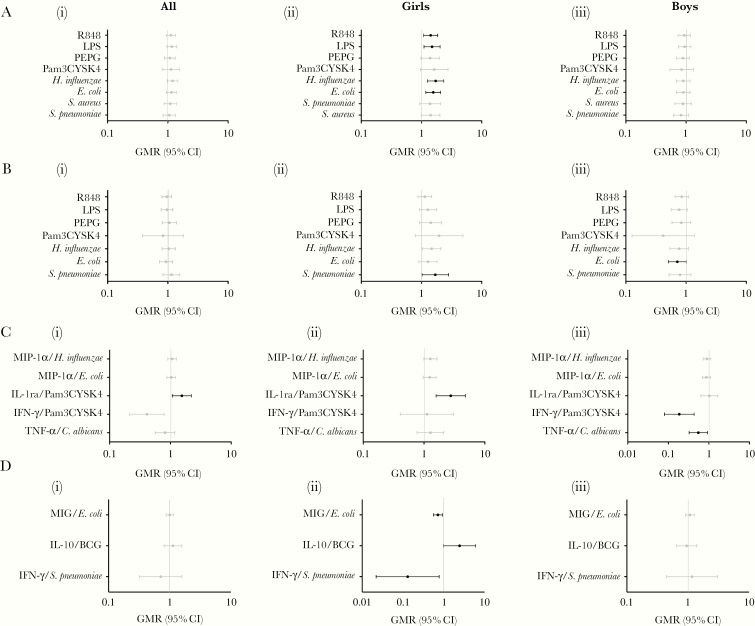
Subgroup analysis for the effect of infant sex and maternal BCG vaccination on in vitro cytokine responses. Interleukin 6 (*A*), interleukin 1β (*B*), and remaining cytokine/stimulant pairs (*C*) with a significant interaction between sex and BCG vaccination. Geometric mean ratios (GMRs) and 95% confidence intervals (CIs) are shown for the effect of infant BCG vaccination on all study participants (i), girls (ii), and boys (iii). *D*, Cytokine/stimulant pairs with a significant interaction between maternal BCG vaccination and BCG vaccination. GMRs and 95% CIs are shown for the effect of infant BCG vaccination on all study participants (i), infants whose mothers were BCG-vaccinated (ii), and infants whose mothers were BCG naive (iii). GMR > 1 indicates that cytokine production is higher in BCG-vaccinated infants. Abbreviations: CI, confidence interval; GMR, geometric mean ratio; IFN, interferon; IL, interleukin; LPS, lipopolysaccharide; MIG, monokine induced by gamma interferon; MIP, macrophage inflammatory protein; PEPG, peptidoglycan; TNF, tumor necrosis factor.

A significant interaction between sex and infant BCG vaccination was seen for multiple cytokine/stimulant pairs, but most consistently for production of IL-6 and IL-1β (Supplementary Table 4). Subgroup analyses of the effect of BCG vaccination in boys and girls separately showed that, compared to BCG-naive girls, BCG-vaccinated girls had increased proinflammatory cytokine production: IL-6 in response to *E. coli*, *H. influenzae*, LPS, and R848; and IL-1β in response to *S. pneumoniae* (Figure 4A and 4B), and an increase in the anti-inflammatory cytokine IL-1Ra in response to Pam3CYSK4 (Figure 4C). Overall, compared to BCG-naive boys, BCG-vaccinated boys had lower cytokine production for all cytokine/stimulant pairs when there was an interaction between infant BCG vaccination and sex. This was statistically significant for IL-1β/*E. coli*, IFN-γ/Pam3CYSK4, and TNF-α/*C. albicans* responses (Figure 4A–C).

Maternal BCG vaccination status independently influenced cytokine responses to heterologous stimulants and TLR ligands. Compared to infants of mothers who had not had BCG vaccination, infants of mothers who were BCG-vaccinated had increased IFN-γ production in response to stimulation with *M. tuberculosis*; decreased IFN-γ in response to PEPG (TLR2/4); decreased TNF-α in response to BCG, *E. coli*, *H. influenzae*, PEPG (TLR2/4), LPS (TLR4), and R848 (TLR7/8); decreased IL-1Ra in response to *H. influenzae*; decreased MCP-1 in response to *S. pneumoniae* and *S. pyogenes*; decreased IP-10 in response to *L. monocytogenes*; and decreased MIP-1β in response to *E. coli* and *S. pneumoniae* (Supplementary Table 1 and Supplementary Figure 3).

A significant interaction between maternal BCG vaccination and infant BCG vaccination was seen for MIG/*E. coli*, IL-10/BCG, and IFN-γ/*S. pneumoniae* cytokine/stimulant pairs (Supplementary Table 4). Separate analyses of the effect of BCG in infants of BCG-vaccinated mothers and infants of BCG-naive mothers showed that in the former there was lower production of MIG in response to *E. coli* and IFN-γ in response to *S. pneumoniae*, whereas BCG vaccination had no effect on this response in infants of BCG-naive mothers (Figure 4D). Conversely, infants of BCG-vaccinated mothers had higher production of IL-10 in response to stimulation with BCG (Figure 4D).

Multivariable analyses were done to evaluate the potential confounding effect of sex, maternal BCG vaccination status, mode of delivery, age at BCG vaccination, age at blood draw, and number of routine vaccination doses received (Supplementary Table 5). Covariate analysis with these variables did not reveal any meaningful effect on the primary analysis, thus excluding confounding by these variables.

## DISCUSSION

We previously reported that BCG vaccination at birth led to decreased production of IL-1ra, IL-6, MCP-1, MIP-1α, and MIP-1β in response to heterologous stimulants and TLR ligands in the neonatal period (7 days after randomization) [11]. This study extends these findings to show that BCG-induced changes in cytokine expression following heterologous stimulation are detectable at 7 months of age, characterized by decreased IFN-γ responsiveness in BCG-vaccinated infants.

Our finding that BCG vaccination was associated with an increase in IFN-γ and proinflammatory cytokines (IL-6, IL-1β, and TNF-α) in response to mycobacterial antigens (BCG and *M. tuberculosis*) is consistent with prior studies [13, 14, 17–19]. Furthermore, our finding that two-thirds of BCG-naive infants produced IFN-γ in response to in vitro stimulation with BCG supports our previous finding of a role for natural killer cells and unconventional T cells in early IFN-γ responses to BCG [20]. Our main finding that neonatal BCG vaccination is associated with a decrease in the proportion of infants who produce IFN-γ in response to heterologous stimulants has not previously been reported.

Nissen et al reported that, in Danish infants at 12 months of age, BCG vaccination did not lead to differential IFN-γ production either in response to ex vivo heterologous bacterial (*E. coli*, *S. pneumoniae*, *C. albicans*) or LPS stimulation [21]. However, in that study approximately half of the BCG-vaccinated participants did not have a specific IFN-γ response to in vitro BCG stimulation. Smith et al also assessed the effects of BCG on in vitro heterologous cytokine responses in infants, although BCG was given at 6 weeks of age [22]. As in our study, BCG-vaccinated infants had an increase in *M. tuberculosis* lysate-induced IFN-γ response. Although there was no significant difference between BCG-vaccinated and BCG-naive infants in IFN-γ production following *E. coli*, *S. aureus*, *C. albicans*, LPS, or Pam3CYSK4 stimulation, fold-change analysis showed decreased LPS-induced IFN-γ responses in BCG-vaccinated infants, an effect that did not reach statistical significance given the small sample size.

Compared to BCG-naive infants, BCG-vaccinated infants in our study showed a distinctive pattern of differential chemokine production that varied between biologically related stimulants. Specifically, in response to mycobacterial, Gram-positive, and intracellular heterologous stimulants, chemokine production in BCG-vaccinated infants was characterized by increased production of MIG and a concomitant decrease in MIP-1α and MIP-1β. MIG is produced by activated monocytes and is the primary chemoattractant for T-helper type 1 cells, which are central to the IFN-γ–dependent proinflammatory response to intracellular bacteria and parasites, and has been recently reported to represent a marker of trained immunity after BCG vaccination [23]. In response to Gram-negative bacteria and LPS, BCG-vaccinated infants had increased IL-8 production. IL-8 is central in the innate response to bacterial infection by promoting neutrophil chemotaxis and macrophage activation. Smith et al reported that, as in our study, BCG-vaccinated infants had significantly higher IL-8 production following stimulation with LPS compared to BCG-naive infants [22]. It remains to be seen whether variable “pathogen-specific” in vitro patterns of innate immune response following BCG vaccination correlate with clinical protection.

For the majority of heterologous stimulants, IFN-γ responses in our study were characterized by a significantly lower proportion of IFN-γ responders in the BCG-vaccinated group, which was responsible for the lower IFN-γ GMR in these infants compared to BCG-naive infants. Variable production of IFN-γ between individuals and between populations is well described [13, 19, 24]. This variability in individual IFN-γ responsiveness, which may be genetically or environmentally determined [25, 26], is a potential modifier of the response to BCG. In a group of BCG-naive, tuberculin skin test–negative adult volunteers in the Netherlands who received BCG vaccination, “nonresponders” were classified as individuals with no IFN-γ response to purified protein derivative and expression of CD8^+^ regulatory T cells, which persisted up to 1 year postvaccination [27]. Fletcher et al reported differential immune responses following BCG vaccination in infants characterized by differences in monocyte:T-cell ratios and an alternatively activated macrophage phenotype (M2) [28]. More recently, Walk et al reported 2 distinct patterns of immune response to human malaria challenge in BCG-vaccinated volunteers. A subgroup of volunteers with strong monocyte and lymphocyte activation were also characterized by increased IFN-γ, granzyme-B production, and C-reactive protein production [29]. This study is the first to report significant changes in IFN-γ responsiveness in the context of BCG-induced heterologous immunity in infants.

IP-10, IL-1Ra, and IL-1β were the only other cytokines that had a subgroup of nonresponders. This is notable in light of the functional relationship of these cytokines to IFN-γ and their role in the control of inflammation and pathogen persistence following mycobacterial infection [30, 31]. The IL-1 signaling axis is central in mediating the response to BCG vaccination in the setting of both mycobacterial and heterologous infections, and IL-1β is a crucial component for the induction of trained immunity [23, 32–34]. However, in our study the proportion of IL-1Ra and IL-1β responders was not significantly different between BCG-vaccinated and BCG-naive infants. Moreover, sensitivity analysis confirmed that increased IL-1Ra responses following stimulation of TLR 1/2 (Pam3CYSK4) seen in BCG-vaccinated infants was unaffected by removal of “nonresponders” from the analysis.

In our previous study, we found that infant sex influenced the effect of BCG vaccination on the production of MIF following heterologous stimulation [11]. In the present study, we observed enhanced *E. coli*, *H. influenzae*, LPS, and R848-induced production of the macrophage derived proinflammatory cytokines IL-6 and IL-1β in BCG-vaccinated girls (in contrast to boys). This is interesting in light of the suggestion that during the neonatal period, the beneficial effects of BCG vaccination on mortality is stronger in boys [35].

Maternal BCG vaccination status is associated with increased BCG scar formation and decreased all-cause mortality in BCG-vaccinated infants [8]. Mawa et al reported altered cytokine responses to TLR ligands, including LPS, in the cord blood of infants of BCG-vaccinated mothers [36]. Our finding that infants of BCG-vaccinated mothers had altered cytokine production following heterologous stimulation, independent of infant BCG vaccination status, raises the possibility that maternal BCG vaccination “primes” the response to infant BCG vaccination. Furthermore, an interaction between maternal and infant BCG vaccination on IFN-γ production in response to *S. pneumoniae* was also found in our previous study; however, maternal BCG had the opposite effect at this time point [11]. This finding is particularly intriguing in light of the finding that BCG vaccine protects infants against respiratory infections only in infants whose mothers were also BCG vaccinated [9, 37].

There is longstanding interest in the variability of BCG vaccine efficacy against tuberculosis infection in different geographic locations. To date, clinically significant heterologous effects of BCG have mainly been documented in African populations [35]. Following BCG vaccination, infant cytokine responses to mycobacteria differ between African and European populations; however, it remains uncertain whether this is due to genetic or environmental influences [13, 14, 38]. In this study, the majority of participants were white, precluding subgroup analysis by ethnicity. Exposure to environmental mycobacteria and chronic helminth infection has been associated with altered BCG-induced IFN-γ production in some populations [38]. It is unlikely that exposure to infection was a confounder in our study, as proxy measures of exposure to common childhood infections (attendance at childcare and number of children aged < 5 years in the household) were similar between groups.

The strengths of this study include its large sample size and the use of samples from participants from a randomized controlled trial. The use of a large number of stimulants and multiplex cytokine analysis enabled a comprehensive interrogation of the immune response. In addition, we used multivariable analysis to identify potential confounders and a highly standardized laboratory protocol to reduce variation. Furthermore, we used the unstimulated sample as a covariate to control for interindividual variability in cytokine responses. A sensitivity analysis, excluding the use of unstimulated samples as a covariate in samples where there was an independent effect on the constitutive expression of a cytokine, showed that this did not lead to overcorrection (data not shown). Our study also has several limitations that are common to research of this type. First, for some cytokine/stimulant pairs, there were lower numbers of participants from whom samples were available. However, this is a large study and our statistical analysis accounted for the varying distributions of individual cytokine/stimulant pair data. Second, while we tested for several potential confounders selected due to their biological plausibility and previously published data, it is possible there are others that may have influenced our findings. Third, there was a risk of type I error due to multiple comparisons. Statistical correction for multiple comparisons was not done as this was an exploratory analysis that aimed to find consistent patterns in BCG-induced alterations in cytokine responses. In addition, we have shown all analyses in their entirety to aid interpretation of the results in context. Finally, routine vaccines from the Australian immunization schedule differ to those administered in low- and middle-income countries.

In summary, at 7 months of age, following neonatal BCG vaccination, infants had lower IFN-γ responses to heterologous stimulants and TLR ligands compared with BCG-naive infants. This resulted from a reduction in the proportion of infants who responded by producing IFN-γ rather than a reduced level of IFN-γ production by all participants. This study is the first to report divergent IFN-γ responses between mycobacterial and heterologous stimulation following BCG vaccination. BCG vaccination is associated with variable patterns of chemokine response depending on the stimulating pathogen. There was a significant interaction between both sex and maternal BCG vaccination with neonatal BCG vaccine-induced responses. Relating the immunological effects of BCG observed among in vitro studies with clinical outcome should be a priority for future research.

## Supplementary Data

Supplementary materials are available at *The Journal of Infectious Diseases* online. Consisting of data provided by the authors to benefit the reader, the posted materials are not copyedited and are the sole responsibility of the authors, so questions or comments should be addressed to the corresponding author.

jiaa030_suppl_Supplementary_Figures_Tables

## Notes

***Acknowledgments.*** The authors thank Judith Spotswood for collection of blood samples; Pedro Ramos for help with processing of blood samples; Casey Goodall for help with data entry; and Dr Laure Pittet for her helpful comments on the manuscript.

***Financial support***. This work was supported by the National Health and Medical Research Council of Australia (NHMRC) (grant numbers GNT1051228 and GNT1099680); the University of Melbourne (International Research Scholarship to B. F.); the European Society of Paediatric Infectious Diseases (fellowship to B. F.); and the Clifford Craig Foundation. M. G. N. was supported by a Spinoza grant of The Netherlands Organization for Scientific Research.

***Potential conflicts of interest.*** All authors: No reported conflicts of interest.
