# Supplementary material for: Neonatal BCG Vaccination Reduces Interferon-γ Responsiveness to Heterologous Pathogens in Infants From a Randomized Controlled Trial
Source: J Infect Dis. 2020 Jan 28;221(12):1999–2009. doi: 10.1093/infdis/jiaa030 (PMC7289544; doi:10.1093/infdis/jiaa030)
Supplement: jiaa030_suppl_Supplementary_Figures_Tables [file jiaa030_suppl_supplementary_figures_tables.docx]

**Neonatal BCG vaccination reduces interferon gamma responsiveness to heterologous pathogens in infants from a randomised controlled trial**

**Supplementary Figure 1**

**Cytokine and chemokine responses to killed heterologous pathogens and Toll-like receptor (TLR) ligands at 7 months of age for all participants**.

Error bars are median (IQR) for each cytokine/stimulant combination.

**Supplementary Figure 2 and Figure 3**

**The effect of sex (Figure 2) and maternal BCG vaccination (Figure 3) on cytokine expression.**

GMR > 1.0 indicates cytokine levels were higher in boys compared with girls (Figure 2) or in infants whose mothers were BCG-vaccinated compared with infants whose mothers were BCG-naïve (Figure 3). Significant results (p<0.05) are depicted in red.

**Supplementary Table 1**

**Univariate analysis of the effect of BCG vaccination, maternal BCG vaccination and sex on neonatal cytokine response to Toll-like receptor ligands and heterologous pathogens.**

% In range refers to the proportion of values that fell within the detectable limit of the assay and were available for statistical analysis. Statistically significant values (p<0.05) are depicted in red. GMR >1.0 indicates the effect was stronger in BCG vaccinated subjects, infants whose mothers’ were BCG-vaccinated and boys. Logistic regression was done with the log-transformed value of the RPMI (nil) cytokine values as a covariate.

* IFN-γ stimulations that were not normally distributed were analysed using quantile regression and the results are depicted as DOM (95% CI).

**Supplementary Table 2**

**Proportion of participants with detectable cytokine production.**

Proportion of BCG-naïve and BCG-vaccinated participants with production of IFN-γ, IP-10, IL-1Ra and IL-1β above the lower limit of detection (‘responders’) in response to heterologous stimulation.

**Supplementary Table 3**

**Sensitivity analysis for the effect of non-responders.**

Sensitivity analysis for the effect of BCG vaccination on the production of IFN-y, IP-10, IL-1β and IL-1Ra after removal of non-responders (all values below the lower limit of detection of the assay) from each stimulation. GMR>1 indicates responses were higher in BCG-vaccinated subjects.

* IFN-γ stimulations that were not normally distributed were analysed using quantile regression and the results are depicted as DOM (95% CI).

**Supplementary Table 4**

**Interaction analysis between infant BCG vaccination and (i) maternal BCG vaccination status and (ii) sex on neonatal cytokine response to Toll-like receptor ligands and heterologous pathogens.**

Statistically significant interactions (p<0.05) are depicted in red, no further results are shown as significant interactions were used as a marker of the appropriateness for further sub-group analysis. Logistic regression was done with the log-transformed value of the RPMI (nil) cytokine values as a covariate. In the absence of a significant interaction no further association is explored.

**Supplementary Table 5**

**Multivariate analysis of the effect of BCG**.

Effect of unstimulated (RPMI) value and individual demographic variables of interest (age at BCG vaccine, age at blood draw, mode of delivery, maternal BCG-vaccination status, sex, number of routine immunisations received) were analysed. Results are depicted as GMR (95% CI) or DOM* (95%CI) and statistically significant results (p<0.05) are marked in red. Logistic regression was done with the log-transformed value of the RPMI (nil) cytokine values as a covariate. The univariate analysis for the effect of BCG vaccination alone is shown in the left-hand column to facilitate comparison with other analyses.

**Abbreviations**

BCG = Bacille Calmette-Guérin, MTBK = killed *Mycobacterium tuberculosis*,

LPS = lipopolysaccharide, PEPG = peptidoglycan, Pam3CYSK4 = (S)-(2,3-bis(palmitoyloxy)-(2-RS)-propyl)-N-palmitoyl-(R)-Cys-(S)-Ser-(S)-Lys4OH,trihydrochloride, R848 = resiquimod,

SA = *S. aureus*, SP = *S. pneumoniae*, GAS = *S. pyogenes*, EC = *E.coli*, HI = *H. influenzae*,

LM = *L. monocytogenes*, CA = *C. albicans*.

DOM = difference in the medians, GMR = geometric mean ratio, IQR = interquartile range, LCI = lower confidence interval, UCI = upper confidence interval.

Supplementary Figure 1, p1


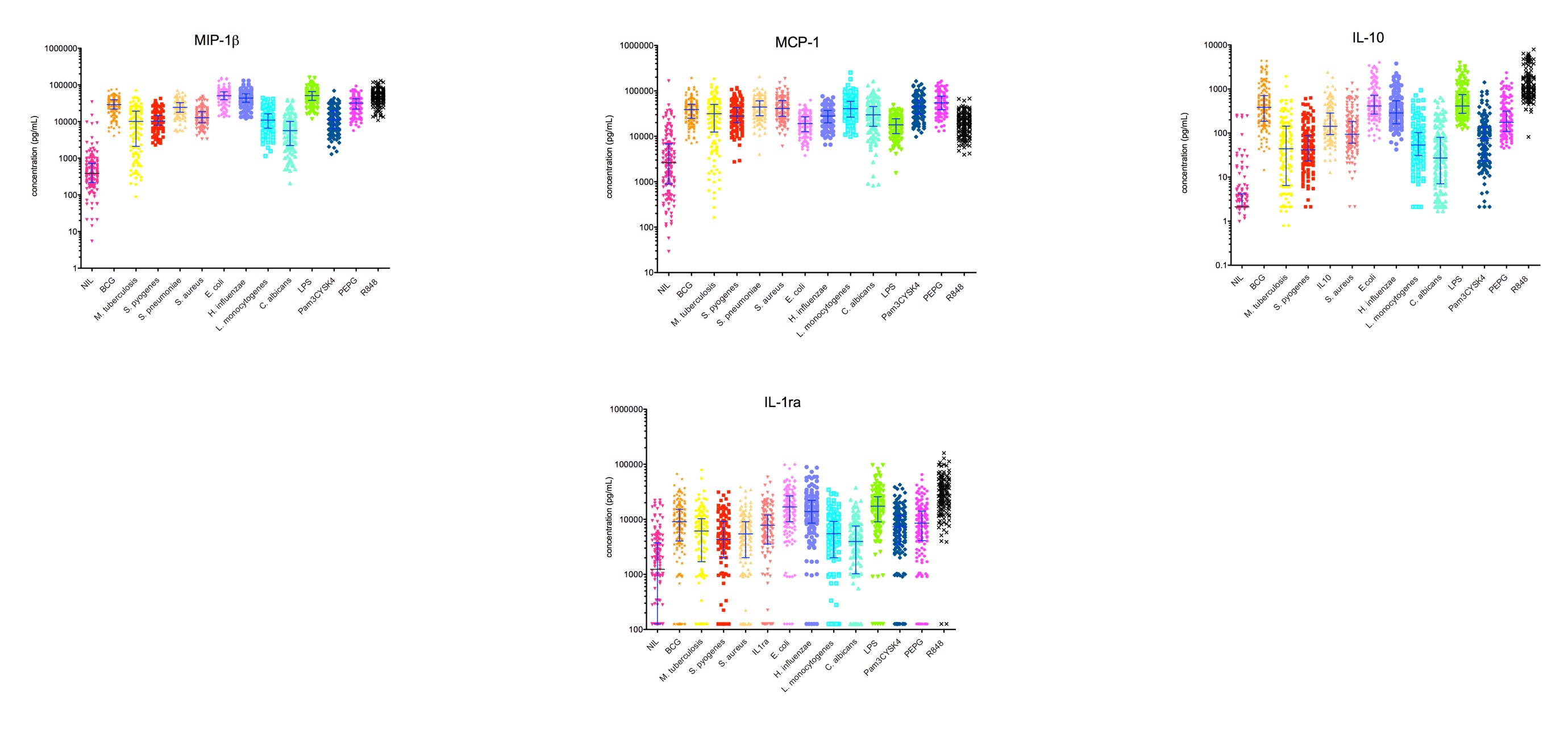


Supplementary Figure 1, p2


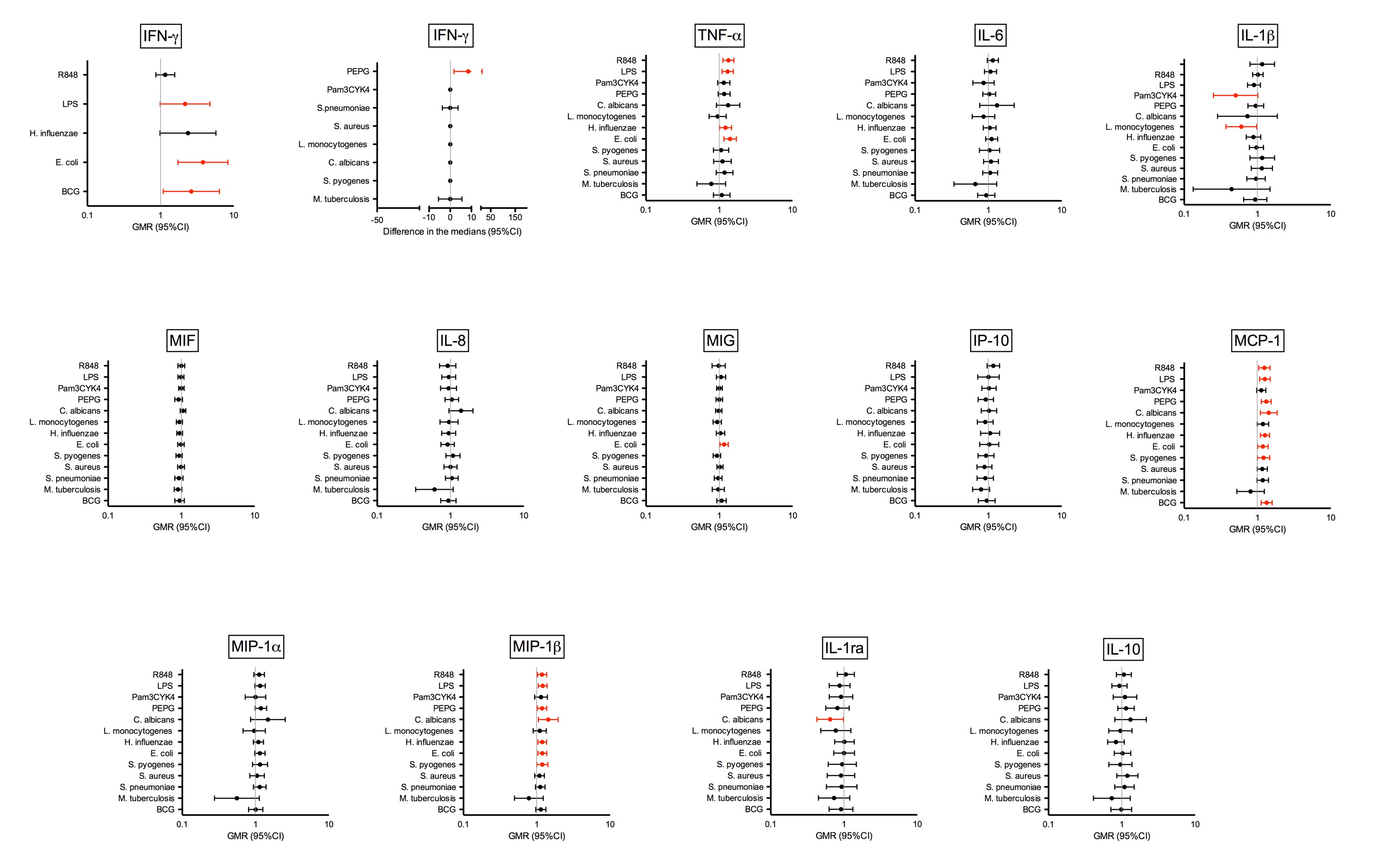


Supplementary Figure 2


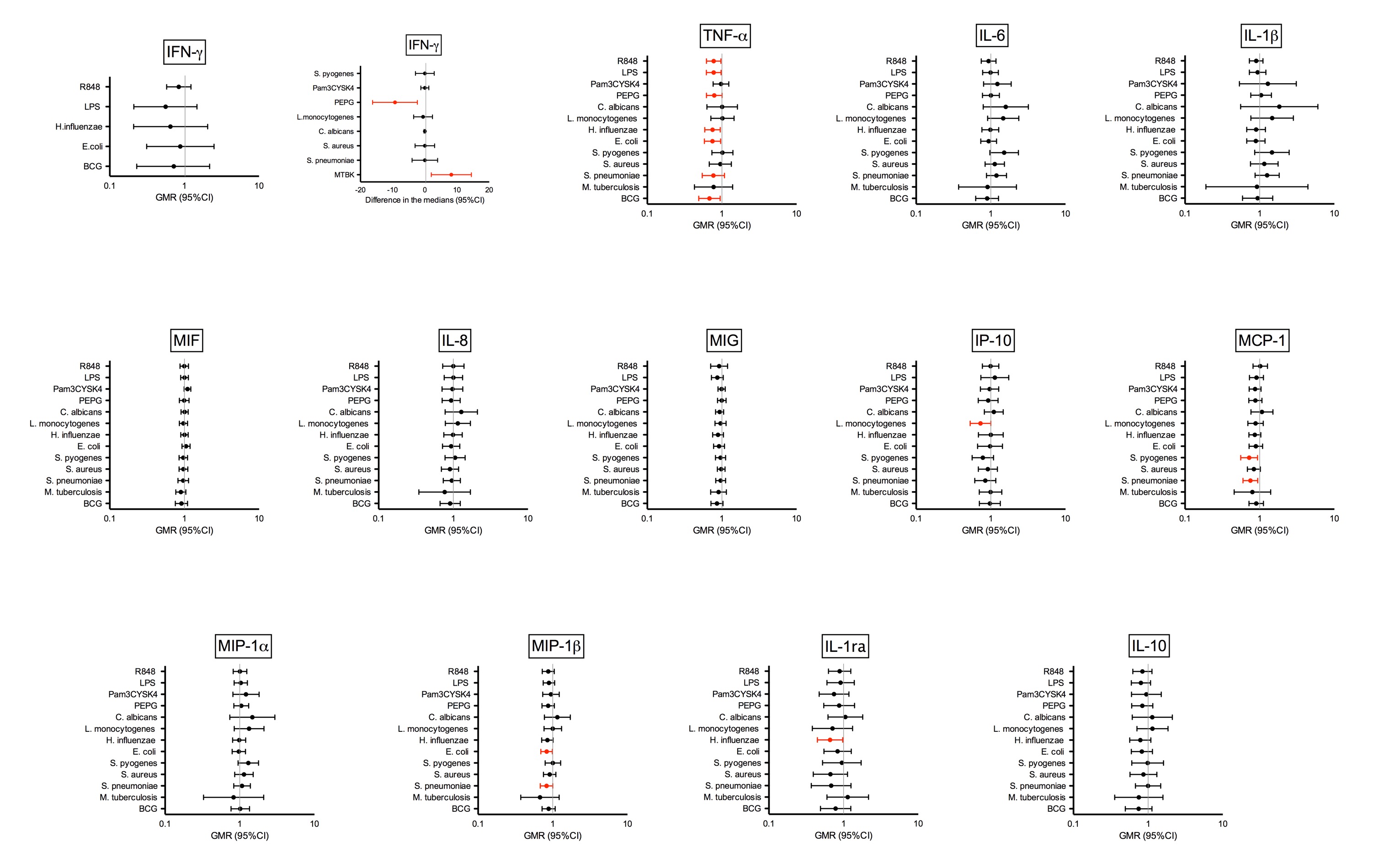
Supplementary Figure 3

|  |  |  | **Neonatal BCG** | | | | **Maternal BCG** | | | | **Sex** | | | |
| --- | --- | --- | --- | --- | --- | --- | --- | --- | --- | --- | --- | --- | --- | --- |
| **Cytokine** | **Stim** | **% in Range** | **GMR** | **95%CI** | | **p-value** | **GMR** | **95%CI** | | **p-value** | **GMR** | **95%CI** | | **p-value** |
|  |  |  |  | **LCI** | **UCI** |  |  | **LCI** | **UCI** |  |  | **LCI** | **UCI** |  |
| **IFN-γ** | BCG | 85.6 | 40.82 | 20.19 | 82.54 | 0.00 | 0.72 | 0.23 | 2.18 | 0.55 | 2.63 | 1.09 | 6.38 | 0.03 |
|  | MTBK* | 53.1 | 8.22 | 1.99 | 14.45 | 0.01 | 8.22 | 1.99 | 14.45 | 0.01 | -5.53 | 0.00 | 5.53 | 1.00 |
|  | SP* | 45.9 | 0.00 | -3.97 | 3.97 | 1.00 | 0.00 | -3.97 | 3.97 | 1.00 | 1.66 | 0.75 | 3.65 | 0.21 |
|  | SA* | 36.1 | 0.00 | -3.02 | 3.02 | 1.00 | 0.00 | -3.03 | 3.03 | 1.00 | 1.36 | 0.68 | 2.70 | 0.38 |
|  | GAS* | 35.2 | 0.00 | -2.90 | 2.90 | 1.00 | 0.00 | -2.91 | 2.91 | 1.00 | 0.00 | 0.00 | 0.00 | 0.91 |
|  | EC | 86.8 | 0.40 | 0.18 | 0.89 | 0.03 | 0.88 | 0.31 | 2.49 | 0.81 | 3.80 | 1.73 | 8.33 | 0.00 |
|  | HI | 77.3 | 0.24 | 0.10 | 0.57 | 0.00 | 0.65 | 0.21 | 2.05 | 0.46 | 2.37 | 0.98 | 5.71 | 0.05 |
|  | LM* | 30.6 | -0.56 | -3.48 | 2.36 | 0.71 | -0.56 | -3.48 | 2.36 | 0.71 | 0.00 | 0.00 | 0.00 | 1.00 |
|  | CA* | 30.7 | 0.00 | -0.23 | 0.23 | 1.00 | 0.00 | -0.23 | 0.23 | 1.00 | 0.00 | 0.00 | 0.00 | 1.00 |
|  | PEPG* | 58.7 | -9.27 | -16.23 | -2.32 | 0.01 | -9.27 | -16.23 | -2.32 | 0.01 | 4.58 | 8.50 | 12.42 | 0.00 |
|  | Pam3* | 34.3 | -1.23 | 0.00 | 1.23 | 1.00 | 0.00 | -1.24 | 1.24 | 1.00 | 0.00 | 0.00 | 0.00 | 0.72 |
|  | LPS | 90.4 | 0.31 | 0.15 | 0.66 | 0.00 | 0.56 | 0.21 | 1.48 | 0.24 | -22.6 | 60.68 | 143.94 | 0.02 |
|  | R848 | 100.0 | 0.78 | 0.58 | 1.05 | 0.10 | 0.84 | 0.58 | 1.23 | 0.37 | 1.16 | 0.86 | 1.56 | 0.32 |
| **TNF-α** | BCG | 100.0 | 1.34 | 1.03 | 1.74 | 0.03 | 0.68 | 0.49 | 0.95 | 0.02 | 1.08 | 0.83 | 1.40 | 0.56 |
|  | MTBK | 100.0 | 1.23 | 0.78 | 1.93 | 0.38 | 0.77 | 0.43 | 1.39 | 0.39 | 0.78 | 0.50 | 1.22 | 0.27 |
|  | SP | 100.0 | 1.04 | 0.80 | 1.36 | 0.76 | 0.77 | 0.54 | 1.08 | 0.13 | 1.18 | 0.91 | 1.54 | 0.21 |
|  | SA | 100.0 | 0.96 | 0.73 | 1.27 | 0.79 | 0.95 | 0.68 | 1.34 | 0.77 | 1.11 | 0.84 | 1.46 | 0.47 |
|  | GAS | 100.0 | 0.96 | 0.75 | 1.22 | 0.72 | 1.01 | 0.73 | 1.40 | 0.93 | 1.06 | 0.83 | 1.35 | 0.64 |
|  | EC | 100.0 | 1.04 | 0.85 | 1.27 | 0.73 | 0.75 | 0.58 | 0.96 | 0.02 | 1.41 | 1.16 | 1.71 | 0.00 |
|  | HI | 100.0 | 1.08 | 0.89 | 1.31 | 0.46 | 0.75 | 0.59 | 0.95 | 0.02 | 1.22 | 1.01 | 1.47 | 0.04 |
|  | LM | 100.0 | 0.83 | 0.63 | 1.09 | 0.17 | 1.02 | 0.71 | 1.45 | 0.93 | 0.95 | 0.73 | 1.25 | 0.71 |
|  | CA | 100.0 | 0.81 | 0.56 | 1.18 | 0.28 | 1.01 | 0.63 | 1.61 | 0.98 | 1.32 | 0.92 | 1.90 | 0.14 |
|  | PEPG | 100.0 | 1.04 | 0.86 | 1.25 | 0.72 | 0.79 | 0.62 | 1.00 | 0.05 | 1.17 | 0.97 | 1.41 | 0.10 |
|  | Pam3 | 100.0 | 1.01 | 0.83 | 1.23 | 0.90 | 0.97 | 0.76 | 1.24 | 0.81 | 1.15 | 0.95 | 1.40 | 0.15 |
|  | LPS | 100.0 | 1.02 | 0.85 | 1.23 | 0.83 | 0.78 | 0.62 | 0.97 | 0.03 | 1.30 | 1.09 | 1.56 | 0.00 |
|  | R848 | 100.0 | 1.11 | 0.93 | 1.33 | 0.24 | 0.78 | 0.62 | 0.97 | 0.03 | 1.33 | 1.12 | 1.58 | 0.00 |
| **IL-6** | BCG | 100.0 | 1.36 | 1.04 | 1.78 | 0.03 | 0.89 | 0.63 | 1.27 | 0.52 | 0.93 | 0.71 | 1.22 | 0.62 |
|  | MTBK | 83.7 | 1.10 | 0.56 | 2.18 | 0.78 | 0.90 | 0.37 | 2.20 | 0.82 | 0.66 | 0.34 | 1.30 | 0.23 |
|  | SP | 100.0 | 1.06 | 0.84 | 1.34 | 0.63 | 1.19 | 0.87 | 1.62 | 0.27 | 1.06 | 0.84 | 1.34 | 0.64 |
|  | GAS | 100.0 | 1.00 | 0.73 | 1.38 | 0.99 | 1.51 | 0.97 | 2.34 | 0.07 | 1.03 | 0.75 | 1.42 | 0.83 |
|  | EC | 100.0 | 1.15 | 0.95 | 1.39 | 0.15 | 0.94 | 0.73 | 1.19 | 0.59 | 1.11 | 0.92 | 1.34 | 0.29 |
|  | HI | 100.0 | 1.20 | 0.98 | 1.47 | 0.07 | 0.99 | 0.76 | 1.28 | 0.91 | 1.04 | 0.85 | 1.27 | 0.70 |
|  | LM | 100.0 | 0.89 | 0.62 | 1.28 | 0.54 | 1.47 | 0.91 | 2.37 | 0.12 | 0.86 | 0.60 | 1.22 | 0.39 |
|  | CA | 94.1 | 0.88 | 0.51 | 1.52 | 0.64 | 1.59 | 0.79 | 3.18 | 0.19 | 1.31 | 0.76 | 2.25 | 0.33 |
|  | PEPG | 100.0 | 1.08 | 0.89 | 1.33 | 0.44 | 1.00 | 0.77 | 1.30 | 0.98 | 1.03 | 0.84 | 1.25 | 0.81 |
|  | Pam3 | 100.0 | 1.13 | 0.81 | 1.58 | 0.48 | 1.22 | 0.80 | 1.87 | 0.35 | 0.86 | 0.61 | 1.20 | 0.36 |
|  | LPS | 100.0 | 1.16 | 0.96 | 1.41 | 0.12 | 0.99 | 0.78 | 1.26 | 0.94 | 1.07 | 0.88 | 1.29 | 0.50 |
|  | R848 | 100.0 | 1.12 | 0.94 | 1.34 | 0.19 | 0.93 | 0.74 | 1.17 | 0.55 | 1.15 | 0.96 | 1.37 | 0.12 |
| **IL-1β** | BCG | 100.0 | 1.12 | 0.75 | 1.67 | 0.58 | 0.94 | 0.59 | 1.50 | 0.79 | 0.93 | 0.65 | 1.34 | 0.70 |
|  | MTBK | 81.9 | 0.80 | 0.21 | 3.07 | 0.75 | 0.92 | 0.19 | 4.43 | 0.92 | 0.44 | 0.13 | 1.48 | 0.19 |
|  | SP | 100.0 | 1.15 | 0.83 | 1.58 | 0.40 | 1.26 | 0.87 | 1.82 | 0.23 | 0.95 | 0.72 | 1.27 | 0.75 |
|  | SA | 99.4 | 0.93 | 0.64 | 1.33 | 0.68 | 1.15 | 0.75 | 1.76 | 0.51 | 1.15 | 0.82 | 1.60 | 0.42 |
|  | GAS | 100.0 | 0.97 | 0.64 | 1.49 | 0.90 | 1.46 | 0.86 | 2.48 | 0.16 | 1.16 | 0.79 | 1.71 | 0.45 |
|  | EC | 100.0 | 0.93 | 0.73 | 1.19 | 0.56 | 0.89 | 0.67 | 1.18 | 0.41 | 0.97 | 0.77 | 1.21 | 0.77 |
|  | HI | 100.0 | 1.03 | 0.80 | 1.33 | 0.80 | 0.90 | 0.67 | 1.19 | 0.45 | 0.88 | 0.70 | 1.11 | 0.28 |
|  | LM | 98.6 | 0.73 | 0.43 | 1.26 | 0.25 | 1.47 | 0.76 | 2.83 | 0.25 | 0.60 | 0.37 | 0.97 | 0.04 |
|  | CA | 85.6 | 1.98 | 0.70 | 5.60 | 0.19 | 1.83 | 0.56 | 6.02 | 0.32 | 0.73 | 0.28 | 1.86 | 0.51 |
|  | PEPG | 100.0 | 1.06 | 0.81 | 1.39 | 0.68 | 1.04 | 0.76 | 1.44 | 0.79 | 0.95 | 0.74 | 1.21 | 0.67 |
|  | Pam3 | 93.4 | 0.83 | 0.38 | 1.80 | 0.64 | 1.29 | 0.53 | 3.10 | 0.57 | 0.50 | 0.25 | 1.01 | 0.05 |
|  | LPS | 100.0 | 0.97 | 0.77 | 1.21 | 0.77 | 0.94 | 0.73 | 1.21 | 0.62 | 0.90 | 0.73 | 1.10 | 0.28 |
|  | R848 | 100.0 | 0.96 | 0.80 | 1.15 | 0.65 | 0.90 | 0.73 | 1.11 | 0.31 | 1.01 | 0.86 | 1.19 | 0.89 |

Supplementary Table 1, p1

|  |  |  | **Neonatal BCG** | | | | **Maternal BCG** | | | | **Sex** | | | | |
| --- | --- | --- | --- | --- | --- | --- | --- | --- | --- | --- | --- | --- | --- | --- | --- |
| **Cytokine** | **Stim** | **% in Range** | **GMR** | **95%CI** | | **p-value** | **GMR** | **95%CI** | | **p-value** | **GMR** | **95%CI** | | **p-value** |  |
|  |  |  |  | **LCI** | **UCI** |  |  | **LCI** | **UCI** |  |  | **LCI** | **UCI** |  |  |
| **MIF** | BCG | 100.0 | 1.05 | 0.90 | 1.22 | 0.54 | 0.91 | 0.76 | 1.10 | 0.33 | 0.94 | 0.81 | 1.09 | 0.43 |  |
|  | MTBK | 100.0 | 1.12 | 0.99 | 1.26 | 0.07 | 0.90 | 0.77 | 1.05 | 0.16 | 0.90 | 0.80 | 1.01 | 0.08 |  |
|  | SP | 100.0 | 1.09 | 0.96 | 1.24 | 0.19 | 0.96 | 0.82 | 1.13 | 0.66 | 0.92 | 0.81 | 1.05 | 0.23 |  |
|  | SA | 100.0 | 1.11 | 0.99 | 1.23 | 0.06 | 0.97 | 0.85 | 1.11 | 0.64 | 0.99 | 0.89 | 1.10 | 0.84 |  |
|  | GAS | 100.0 | 1.11 | 1.01 | 1.22 | 0.04 | 0.97 | 0.85 | 1.10 | 0.60 | 0.93 | 0.84 | 1.02 | 0.14 |  |
|  | EC | 100.0 | 1.10 | 0.99 | 1.22 | 0.06 | 1.05 | 0.93 | 1.19 | 0.44 | 0.99 | 0.89 | 1.09 | 0.78 |  |
|  | HI | 100.0 | 1.09 | 0.99 | 1.20 | 0.07 | 1.00 | 0.89 | 1.12 | 0.97 | 0.94 | 0.86 | 1.04 | 0.22 |  |
|  | LM | 100.0 | 1.05 | 0.95 | 1.15 | 0.35 | 0.97 | 0.86 | 1.10 | 0.63 | 0.94 | 0.85 | 1.03 | 0.19 |  |
|  | CA | 100.0 | 1.05 | 0.97 | 1.15 | 0.24 | 1.01 | 0.90 | 1.12 | 0.92 | 1.05 | 0.96 | 1.15 | 0.26 |  |
|  | PEPG | 100.0 | 1.07 | 0.94 | 1.20 | 0.31 | 0.99 | 0.86 | 1.15 | 0.92 | 0.92 | 0.81 | 1.03 | 0.15 |  |
|  | Pam3 | 100.0 | 1.06 | 0.98 | 1.16 | 0.16 | 1.10 | 0.99 | 1.22 | 0.07 | 1.00 | 0.92 | 1.09 | 0.97 |  |
|  | LPS | 100.0 | 1.08 | 0.98 | 1.19 | 0.10 | 1.00 | 0.89 | 1.13 | 0.97 | 0.98 | 0.90 | 1.08 | 0.75 |  |
|  | R848 | 100.0 | 1.10 | 0.99 | 1.22 | 0.09 | 0.99 | 0.87 | 1.13 | 0.93 | 1.00 | 0.89 | 1.11 | 0.94 |  |
| **MIG** | BCG | 100.0 | 1.48 | 1.29 | 1.69 | 0.00 | 0.86 | 0.71 | 1.03 | 0.10 | 1.07 | 0.92 | 1.24 | 0.36 |  |
|  | MTBK | 100.0 | 1.44 | 1.20 | 1.72 | 0.00 | 0.90 | 0.71 | 1.15 | 0.39 | 0.97 | 0.80 | 1.17 | 0.72 |  |
|  | SP | 100.0 | 1.12 | 0.99 | 1.26 | 0.07 | 0.96 | 0.82 | 1.12 | 0.61 | 0.96 | 0.85 | 1.09 | 0.54 |  |
|  | SA | 100.0 | 1.11 | 1.01 | 1.22 | 0.02 | 0.98 | 0.87 | 1.10 | 0.69 | 1.02 | 0.93 | 1.12 | 0.68 |  |
|  | GAS | 100.0 | 1.13 | 1.01 | 1.26 | 0.04 | 0.96 | 0.82 | 1.12 | 0.59 | 0.93 | 0.82 | 1.04 | 0.20 |  |
|  | EC | 100.0 | 1.00 | 0.87 | 1.14 | 0.96 | 0.91 | 0.77 | 1.08 | 0.30 | 1.16 | 1.02 | 1.33 | 0.03 |  |
|  | HI | 100.0 | 1.06 | 0.93 | 1.21 | 0.36 | 0.89 | 0.75 | 1.05 | 0.16 | 1.04 | 0.91 | 1.18 | 0.59 |  |
|  | LM | 99.3 | 1.11 | 0.98 | 1.27 | 0.11 | 0.96 | 0.81 | 1.14 | 0.62 | 0.94 | 0.82 | 1.07 | 0.32 |  |
|  | CA | 100.0 | 1.11 | 1.01 | 1.23 | 0.03 | 0.93 | 0.82 | 1.05 | 0.26 | 0.98 | 0.89 | 1.08 | 0.70 |  |
|  | PEPG | 99.4 | 1.05 | 0.95 | 1.17 | 0.34 | 1.00 | 0.87 | 1.13 | 0.94 | 1.00 | 0.90 | 1.11 | 0.98 |  |
|  | Pam3 | 100.0 | 1.08 | 0.99 | 1.19 | 0.07 | 0.99 | 0.89 | 1.11 | 0.85 | 1.00 | 0.92 | 1.10 | 0.95 |  |
|  | LPS | 99.4 | 1.03 | 0.89 | 1.19 | 0.70 | 0.87 | 0.72 | 1.04 | 0.13 | 1.05 | 0.91 | 1.22 | 0.47 |  |
|  | R848 | 100.0 | 0.98 | 0.80 | 1.21 | 0.86 | 0.92 | 0.71 | 1.19 | 0.51 | 0.98 | 0.80 | 1.20 | 0.83 |  |
| **MCP-1** | BCG | 100.0 | 0.96 | 0.81 | 1.15 | 0.68 | 0.90 | 0.72 | 1.12 | 0.35 | 1.33 | 1.12 | 1.58 | 0.00 |  |
|  | MTBK | 100.0 | 1.04 | 0.67 | 1.60 | 0.87 | 0.80 | 0.45 | 1.40 | 0.43 | 0.80 | 0.52 | 1.24 | 0.32 |  |
|  | SP | 100.0 | 0.93 | 0.77 | 1.11 | 0.42 | 0.75 | 0.60 | 0.94 | 0.01 | 1.18 | 0.99 | 1.41 | 0.07 |  |
|  | SA | 100.0 | 0.94 | 0.80 | 1.11 | 0.48 | 0.84 | 0.68 | 1.02 | 0.08 | 1.16 | 0.99 | 1.36 | 0.06 |  |
|  | GAS | 100.0 | 0.94 | 0.77 | 1.14 | 0.52 | 0.72 | 0.56 | 0.94 | 0.01 | 1.21 | 1.00 | 1.47 | 0.05 |  |
|  | EC | 100.0 | 0.96 | 0.81 | 1.13 | 0.60 | 0.89 | 0.72 | 1.09 | 0.27 | 1.19 | 1.01 | 1.40 | 0.04 |  |
|  | HI | 100.0 | 0.94 | 0.81 | 1.09 | 0.44 | 0.86 | 0.72 | 1.03 | 0.11 | 1.26 | 1.10 | 1.46 | 0.00 |  |
|  | LM | 100.0 | 0.91 | 0.76 | 1.09 | 0.29 | 0.88 | 0.69 | 1.12 | 0.29 | 1.19 | 0.99 | 1.42 | 0.06 |  |
|  | CA | 100.0 | 0.89 | 0.68 | 1.16 | 0.38 | 1.07 | 0.76 | 1.51 | 0.68 | 1.42 | 1.10 | 1.85 | 0.01 |  |
|  | PEPG | 100.0 | 0.97 | 0.83 | 1.14 | 0.75 | 0.88 | 0.72 | 1.07 | 0.19 | 1.31 | 1.13 | 1.53 | 0.00 |  |
|  | Pam3 | 100.0 | 1.00 | 0.86 | 1.16 | 0.99 | 0.87 | 0.72 | 1.04 | 0.13 | 1.13 | 0.97 | 1.30 | 0.18 |  |
|  | LPS | 100.0 | 0.97 | 0.82 | 1.15 | 0.71 | 0.90 | 0.73 | 1.12 | 0.36 | 1.26 | 1.07 | 1.49 | 0.01 |  |
|  | R848 | 100.0 | 0.99 | 0.83 | 1.19 | 0.91 | 1.02 | 0.81 | 1.27 | 0.88 | 1.24 | 1.04 | 1.48 | 0.02 |  |
| **IL-8** | BCG | 100.0 | 1.21 | 0.95 | 1.54 | 0.12 | 0.91 | 0.66 | 1.24 | 0.54 | 0.94 | 0.74 | 1.19 | 0.58 |  |
|  | MTBK | 99.3 | 1.07 | 0.59 | 1.96 | 0.82 | 0.77 | 0.35 | 1.70 | 0.51 | 0.60 | 0.34 | 1.09 | 0.09 |  |
|  | SP | 100.0 | 1.04 | 0.85 | 1.27 | 0.70 | 0.95 | 0.73 | 1.24 | 0.73 | 1.04 | 0.85 | 1.27 | 0.70 |  |
|  | SA | 100.0 | 1.07 | 0.87 | 1.31 | 0.53 | 0.90 | 0.69 | 1.18 | 0.44 | 1.00 | 0.82 | 1.23 | 0.99 |  |
|  | GAS | 100.0 | 1.09 | 0.87 | 1.37 | 0.46 | 1.06 | 0.78 | 1.44 | 0.73 | 1.08 | 0.87 | 1.35 | 0.49 |  |
|  | EC | 100.0 | 1.23 | 1.00 | 1.52 | 0.05 | 0.94 | 0.72 | 1.22 | 0.63 | 0.91 | 0.74 | 1.12 | 0.39 |  |
|  | HI | 100.0 | 1.23 | 0.98 | 1.53 | 0.07 | 0.99 | 0.75 | 1.32 | 0.95 | 0.94 | 0.76 | 1.18 | 0.61 |  |
|  | LM | 100.0 | 0.95 | 0.71 | 1.26 | 0.70 | 1.15 | 0.78 | 1.69 | 0.47 | 0.95 | 0.72 | 1.27 | 0.75 |  |
|  | CA | 100.0 | 0.92 | 0.62 | 1.35 | 0.65 | 1.28 | 0.78 | 2.10 | 0.33 | 1.39 | 0.95 | 2.03 | 0.09 |  |
|  | PEPG | 100.0 | 1.04 | 0.84 | 1.29 | 0.74 | 0.94 | 0.71 | 1.23 | 0.63 | 1.04 | 0.84 | 1.29 | 0.71 |  |
|  | Pam3 | 100.0 | 1.13 | 0.88 | 1.46 | 0.34 | 0.97 | 0.71 | 1.34 | 0.87 | 0.94 | 0.73 | 1.21 | 0.63 |  |
|  | LPS | 100.0 | 1.29 | 1.03 | 1.60 | 0.02 | 1.00 | 0.76 | 1.33 | 0.98 | 0.95 | 0.76 | 1.18 | 0.62 |  |
|  | R848 | 100.0 | 1.14 | 0.88 | 1.48 | 0.31 | 1.00 | 0.72 | 1.39 | 0.99 | 0.91 | 0.71 | 1.18 | 0.49 |  |

Supplementary Table 1, p2

|  |  |  | **Neonatal BCG** | | | | **Maternal BCG** | | | | **Sex** | | | | |
| --- | --- | --- | --- | --- | --- | --- | --- | --- | --- | --- | --- | --- | --- | --- | --- |
| **Cytokine** | **Stim** | **% in Range** | **GMR** | **95%CI** | | **p-value** | **GMR** | **95%CI** | | **p-value** | **GMR** | **95%CI** | | | **p-value** |
|  |  |  |  | **LCI** | **UCI** |  |  | **LCI** | **UCI** |  |  | **LCI** | **UCI** | |  |
| **IP-10** | BCG | 60.8 | 1.18 | 0.90 | 1.55 | 0.23 | 0.97 | 0.70 | 1.34 | 0.85 | 0.95 | 0.73 | | 1.24 | 0.69 |
|  | MTBK | 49.7 | 0.96 | 0.73 | 1.27 | 0.77 | 0.99 | 0.70 | 1.40 | 0.96 | 0.79 | 0.61 | | 1.04 | 0.09 |
|  | SP | 100.0 | 1.02 | 0.78 | 1.32 | 0.89 | 0.84 | 0.61 | 1.17 | 0.30 | 0.91 | 0.70 | | 1.17 | 0.45 |
|  | SA | 100.0 | 0.91 | 0.72 | 1.16 | 0.47 | 0.91 | 0.68 | 1.22 | 0.54 | 0.88 | 0.70 | | 1.11 | 0.29 |
|  | GAS | 51.4 | 0.98 | 0.76 | 1.27 | 0.89 | 0.78 | 0.56 | 1.09 | 0.14 | 0.93 | 0.72 | | 1.19 | 0.55 |
|  | EC | 74.8 | 0.92 | 0.67 | 1.25 | 0.58 | 0.98 | 0.67 | 1.43 | 0.90 | 1.02 | 0.76 | | 1.38 | 0.88 |
|  | HI | 65.9 | 0.95 | 0.70 | 1.29 | 0.74 | 1.00 | 0.68 | 1.46 | 1.00 | 1.05 | 0.78 | | 1.42 | 0.73 |
|  | LM | 52.1 | 0.91 | 0.71 | 1.18 | 0.48 | 0.73 | 0.53 | 1.00 | 0.05 | 0.91 | 0.71 | | 1.16 | 0.43 |
|  | CA | 51.0 | 0.93 | 0.72 | 1.19 | 0.56 | 1.10 | 0.82 | 1.47 | 0.53 | 1.02 | 0.80 | | 1.30 | 0.89 |
|  | PEPG | 100.0 | 0.94 | 0.73 | 1.20 | 0.60 | 0.92 | 0.68 | 1.25 | 0.60 | 0.92 | 0.72 | | 1.17 | 0.50 |
|  | Pam3 | 100.0 | 1.00 | 0.79 | 1.26 | 0.98 | 0.96 | 0.73 | 1.27 | 0.79 | 1.02 | 0.81 | | 1.27 | 0.87 |
|  | LPS | 77.2 | 0.86 | 0.61 | 1.22 | 0.41 | 1.13 | 0.73 | 1.74 | 0.57 | 1.00 | 0.72 | | 1.40 | 0.99 |
|  | R848 | 100.0 | 1.00 | 0.82 | 1.23 | 0.98 | 0.99 | 0.77 | 1.28 | 0.95 | 1.17 | 0.96 | | 1.42 | 0.12 |
| **MIP1-α** | BCG | 100.0 | 1.15 | 0.92 | 1.44 | 0.21 | 1.02 | 0.76 | 1.35 | 0.92 | 1.00 | 0.80 | | 1.26 | 0.97 |
|  | MTBK | 100.0 | 0.95 | 0.47 | 1.96 | 0.90 | 0.83 | 0.33 | 2.09 | 0.69 | 0.56 | 0.28 | | 1.12 | 0.10 |
|  | SP | 100.0 | 0.93 | 0.76 | 1.13 | 0.45 | 1.07 | 0.83 | 1.39 | 0.58 | 1.14 | 0.94 | | 1.39 | 0.19 |
|  | SA | 100.0 | 0.84 | 0.67 | 1.06 | 0.13 | 1.14 | 0.86 | 1.51 | 0.37 | 1.05 | 0.83 | | 1.32 | 0.69 |
|  | GAS | 100.0 | 0.90 | 0.71 | 1.14 | 0.37 | 1.30 | 0.95 | 1.78 | 0.10 | 1.15 | 0.91 | | 1.46 | 0.24 |
|  | EC | 100.0 | 1.03 | 0.87 | 1.21 | 0.74 | 0.97 | 0.79 | 1.19 | 0.78 | 1.15 | 0.98 | | 1.35 | 0.10 |
|  | HI | 100.0 | 1.06 | 0.91 | 1.25 | 0.44 | 0.98 | 0.80 | 1.20 | 0.86 | 1.09 | 0.93 | | 1.28 | 0.27 |
|  | LM | 100.0 | 0.72 | 0.51 | 1.02 | 0.06 | 1.33 | 0.84 | 2.11 | 0.22 | 0.96 | 0.68 | | 1.36 | 0.81 |
|  | CA | 100.0 | 0.73 | 0.42 | 1.26 | 0.25 | 1.47 | 0.74 | 2.95 | 0.27 | 1.48 | 0.86 | | 2.54 | 0.16 |
|  | PEPG | 100.0 | 0.92 | 0.77 | 1.10 | 0.35 | 1.05 | 0.83 | 1.32 | 0.68 | 1.18 | 0.99 | | 1.41 | 0.06 |
|  | Pam3 | 100.0 | 1.05 | 0.76 | 1.45 | 0.78 | 1.21 | 0.81 | 1.81 | 0.35 | 1.00 | 0.72 | | 1.38 | 0.99 |
|  | LPS | 100.0 | 1.00 | 0.85 | 1.19 | 0.96 | 1.03 | 0.84 | 1.26 | 0.76 | 1.15 | 0.98 | | 1.36 | 0.09 |
|  | R848 | 100.0 | 1.01 | 0.85 | 1.19 | 0.94 | 1.01 | 0.82 | 1.24 | 0.95 | 1.12 | 0.95 | | 1.32 | 0.18 |
| **MIP-1β** | BCG | 100.0 | 0.95 | 0.81 | 1.11 | 0.51 | 0.88 | 0.72 | 1.08 | 0.22 | 1.14 | 0.97 | | 1.33 | 0.12 |
|  | MTBK | 100.0 | 1.05 | 0.66 | 1.65 | 0.84 | 0.67 | 0.37 | 1.22 | 0.19 | 0.78 | 0.50 | | 1.23 | 0.28 |
|  | SP | 100.0 | 0.91 | 0.78 | 1.05 | 0.20 | 0.83 | 0.68 | 1.00 | 0.05 | 1.12 | 0.96 | | 1.30 | 0.15 |
|  | SA | 100.0 | 0.86 | 0.74 | 1.00 | 0.05 | 0.91 | 0.75 | 1.10 | 0.32 | 1.09 | 0.93 | | 1.27 | 0.27 |
|  | GAS | 100.0 | 0.90 | 0.75 | 1.07 | 0.21 | 1.00 | 0.79 | 1.27 | 0.98 | 1.19 | 1.00 | | 1.42 | 0.05 |
|  | EC | 100.0 | 0.98 | 0.85 | 1.13 | 0.80 | 0.83 | 0.69 | 0.99 | 0.04 | 1.19 | 1.04 | | 1.37 | 0.01 |
|  | HI | 100.0 | 0.99 | 0.86 | 1.14 | 0.91 | 0.85 | 0.71 | 1.01 | 0.06 | 1.19 | 1.04 | | 1.36 | 0.01 |
|  | LM | 100.0 | 0.80 | 0.65 | 0.98 | 0.03 | 1.00 | 0.76 | 1.31 | 1.00 | 1.10 | 0.89 | | 1.35 | 0.38 |
|  | CA | 100.0 | 0.78 | 0.57 | 1.06 | 0.11 | 1.15 | 0.77 | 1.72 | 0.48 | 1.43 | 1.05 | | 1.96 | 0.02 |
|  | PEPG | 100.0 | 0.90 | 0.78 | 1.05 | 0.19 | 0.87 | 0.72 | 1.05 | 0.14 | 1.18 | 1.02 | | 1.37 | 0.03 |
|  | Pam3 | 100.0 | 0.93 | 0.76 | 1.13 | 0.46 | 0.95 | 0.73 | 1.22 | 0.68 | 1.14 | 0.93 | | 1.39 | 0.19 |
|  | LPS | 100.0 | 0.98 | 0.85 | 1.13 | 0.78 | 0.89 | 0.74 | 1.06 | 0.19 | 1.20 | 1.05 | | 1.38 | 0.01 |
|  | R848 | 100.0 | 0.98 | 0.85 | 1.14 | 0.82 | 0.87 | 0.72 | 1.05 | 0.14 | 1.18 | 1.02 | | 1.36 | 0.03 |
| **IL-10** | BCG | 100.0 | 1.13 | 0.81 | 1.57 | 0.47 | 0.75 | 0.50 | 1.13 | 0.16 | 0.98 | 0.71 | | 1.36 | 0.91 |
|  | MTBK | 100.0 | 1.02 | 0.56 | 1.86 | 0.94 | 0.75 | 0.36 | 1.58 | 0.45 | 0.73 | 0.41 | | 1.30 | 0.28 |
|  | SP | 100.0 | 1.06 | 0.77 | 1.45 | 0.72 | 1.00 | 0.68 | 1.47 | 0.99 | 1.09 | 0.80 | | 1.47 | 0.59 |
|  | SA | 100.0 | 0.94 | 0.67 | 1.31 | 0.71 | 0.86 | 0.57 | 1.31 | 0.49 | 1.19 | 0.86 | | 1.66 | 0.30 |
|  | GAS | 100.0 | 0.94 | 0.65 | 1.37 | 0.75 | 0.99 | 0.60 | 1.61 | 0.95 | 0.96 | 0.66 | | 1.38 | 0.81 |
|  | EC | 100.0 | 1.00 | 0.77 | 1.31 | 0.97 | 0.82 | 0.60 | 1.14 | 0.24 | 1.02 | 0.79 | | 1.32 | 0.87 |
|  | HI | 100.0 | 1.07 | 0.82 | 1.40 | 0.63 | 0.79 | 0.57 | 1.09 | 0.14 | 0.83 | 0.64 | | 1.08 | 0.17 |
|  | LM | 100.0 | 0.67 | 0.46 | 0.97 | 0.04 | 1.14 | 0.71 | 1.84 | 0.58 | 0.96 | 0.66 | | 1.38 | 0.81 |
|  | CA | 100.0 | 0.92 | 0.56 | 1.54 | 0.76 | 1.14 | 0.61 | 2.11 | 0.68 | 1.31 | 0.80 | | 2.15 | 0.28 |
|  | PEPG | 100.0 | 1.00 | 0.77 | 1.30 | 0.99 | 0.84 | 0.60 | 1.16 | 0.28 | 1.14 | 0.88 | | 1.48 | 0.31 |
|  | Pam3 | 100.0 | 1.01 | 0.69 | 1.47 | 0.97 | 0.95 | 0.60 | 1.50 | 0.82 | 1.10 | 0.76 | | 1.60 | 0.59 |
|  | LPS | 100.0 | 1.02 | 0.80 | 1.30 | 0.90 | 0.80 | 0.59 | 1.08 | 0.14 | 0.93 | 0.73 | | 1.18 | 0.54 |
|  | R848 | 100.0 | 0.97 | 0.76 | 1.23 | 0.80 | 0.84 | 0.62 | 1.12 | 0.24 | 1.07 | 0.84 | | 1.35 | 0.59 |
| **IL-1ra** | BCG | 91.7 | 1.07 | 0.73 | 1.56 | 0.73 | 0.78 | 0.49 | 1.25 | 0.30 | 0.91 | 0.63 | | 1.32 | 0.62 |
|  | MTBK | 83.7 | 1.45 | 0.88 | 2.38 | 0.14 | 1.14 | 0.60 | 2.16 | 0.69 | 0.73 | 0.45 | | 1.20 | 0.21 |
|  | SP | 87.0 | 1.24 | 0.77 | 2.01 | 0.38 | 0.68 | 0.37 | 1.26 | 0.22 | 0.93 | 0.57 | | 1.50 | 0.76 |
|  | SA | 84.8 | 1.14 | 0.74 | 1.76 | 0.55 | 0.67 | 0.39 | 1.13 | 0.13 | 0.91 | 0.59 | | 1.40 | 0.66 |
|  | GAS | 84.5 | 1.22 | 0.78 | 1.89 | 0.38 | 0.95 | 0.52 | 1.72 | 0.86 | 0.94 | 0.61 | | 1.47 | 0.80 |
|  | EC | 97.6 | 1.10 | 0.79 | 1.52 | 0.58 | 0.83 | 0.55 | 1.27 | 0.39 | 1.00 | 0.72 | | 1.39 | 1.00 |
|  | HI | 97.0 | 1.18 | 0.87 | 1.61 | 0.29 | 0.66 | 0.45 | 0.97 | 0.04 | 1.01 | 0.74 | | 1.38 | 0.94 |
|  | LM | 84.7 | 1.37 | 0.85 | 2.20 | 0.20 | 0.71 | 0.38 | 1.32 | 0.28 | 0.77 | 0.48 | | 1.24 | 0.28 |
|  | CA | 83.7 | 1.16 | 0.76 | 1.78 | 0.48 | 1.06 | 0.62 | 1.81 | 0.84 | 0.65 | 0.43 | | 0.98 | 0.04 |
|  | PEPG | 92.8 | 0.87 | 0.60 | 1.26 | 0.45 | 0.87 | 0.54 | 1.40 | 0.57 | 0.81 | 0.56 | | 1.17 | 0.27 |
|  | Pam3 | 90.4 | 1.54 | 1.07 | 2.22 | 0.02 | 0.75 | 0.47 | 1.18 | 0.21 | 0.91 | 0.63 | | 1.31 | 0.61 |
|  | LPS | 97.0 | 1.01 | 0.72 | 1.40 | 0.97 | 0.91 | 0.60 | 1.39 | 0.67 | 0.87 | 0.63 | | 1.21 | 0.41 |
|  | R848 | 98.8 | 1.03 | 0.79 | 1.35 | 0.82 | 0.89 | 0.63 | 1.25 | 0.49 | 1.06 | 0.81 | | 1.38 | 0.69 |

Supplementary Table 1, p3

|  | **IFN-γ** | | | **IP-10** | | | **IL-1Ra** | | | | **IL-1β** | | |
| --- | --- | --- | --- | --- | --- | --- | --- | --- | --- | --- | --- | --- | --- |
| **Stimulant** | **BCG naïve** | **BCG vaccinated** | **p-value** | **BCG naïve** | **BCG vaccinated** | **p-value** | | **BCG naïve** | **BCG vaccinated** | **p-value** | **BCG naïve** | **BCG vaccinated** | **p-value** |
| **BCG** | 46/68 (67.6) | 97/99 (98.0) | **<0.0001** | 40/69 (58.0) | 59/99 (59.6) | 0.87 | | 64/69 (93.0) | 91/99 (92.0) | 1.00 | 68/68 (100.0) | 99/99 (100.0) | - |
| ***M. tuberculosis*** | 24/59 (40.7) | 54/88 (61.4) | **0.02** | 33/59 (55.9) | 41/88 (46.6) | 0.31 | | 37/59 (62.7) | 77/88 (87.5) | 0.25 | 52/59 (88.1) | 67/88 (76.1) | 0.09 |
| ***E. coli*** | 64/68 (94.1) | 81/99 (81.8) | **0.02** | 55/68 (80.9) | 71/99 (71.7) | 0.21 | | 66/68 (97.1) | 97/99 (98.0) | 1.00 | 68/68 (100.0) | 99/99 (100.0) | - |
| ***H. influenzae*** | 62/68 (91.2) | 67/99 (67.7) | **0.0003** | 40/68 (58.8) | 61/99 (61.6) | 0.25 | | 66/68 (97.1) | 96/99 (97.0) | 1.00 | 68/68 (100.0) | 99/99 (100.0) | - |
| ***L. monocytogenes*** | 30/57 (52.6) | 15/87 (17.2) | **<0.0001** | 32/57 (56.1) | 45/87 (51.7) | 0.61 | | 45/57 (78.9) | 77/87 (88.5) | 0.24 | 57/57 (100.0) | 85/87 (97.7) | 0.51 |
| ***C. albicans*** | 27/62 (43.5) | 19/91 (20.9) | **0.0071** | 35/62 (56.4) | 42/91 (46.2) | 0.25 | | 50/62 (80.6) | 78/91 (85.7) | 0.50 | 52/62 (83.9) | 79/91 (86.8) | 0.64 |
| ***S. aureus*** | 32/66 (48.5) | 26/92 (28.3) | **0.01** | 35/66 (53.0) | 40/90 (44.4) | 0.33 | | 56/66 (84.8) | 80/92 (87.0) | 0.81 | 66/66 (100.0) | 91/92 (98.9) | 1.00 |
| ***S. pneumoniae*** | 29/58 (50.0) | 38/88 (43.2) | 0.49 | 30/58 (51.7) | 47/88 (53.4) | 0.87 | | 50/58 (86.2) | 78/88 (88.6) | 0.80 | 58/58 (100.0) | 88/88 (100.0) | - |
| ***S. pyogenes*** | 26/57 (45.6) | 24/85 (28.2) | **0.05** | 31/57 (54.4) | 42/85 (49.4) | 0.61 | | 47/57 (82.5) | 76/87 (87.4) | 0.47 | 57/57 (100.0) | 84/85 (98.8) | 1.00 |
| **Pam3CYSK4** | 35/68 (51.5) | 25/98 (25.5) | **0.0009** | 36/68 (52.9) | 47/98 (48.0) | 0.53 | | 59/68 (86.8) | 91/98 (93.0) | 0.28 | 66/68 (97.1) | 89/98 (90.8) | 0.20 |
| **PEPG** | 51/68 (75.0) | 47/99 (47.5) | **0.0004** | 41/69 (59.4) | 53/99 (53.5) | 0.42 | | 66/68 (97.1) | 90/99 (91.1) | 0.20 | 68/68 (100.0) | 99/99 (100.0) | - |
| **LPS** | 68/68 (100.0) | 83/99 (83.8) | **<0.0001** | 56/68 (82.4) | 73/99 (73.7) | 0.56 | | 66/68 (97.1) | 96/99 (97.0) | 1.00 | 68/68 (100.0) | 99/99 (100.0) | - |
| **R848** | 68/68 (100.0) | 99/99 (100.0) | 1.00 | 99/99 (100.0) | 68/68 (100) | 1.00 | | 68/68 (100) | 97/99 (98.0) | 1.00 | 68/68 (100.0) | 99/99 (100.0) | - |

Supplementary Table 2

Supplementary Table 3


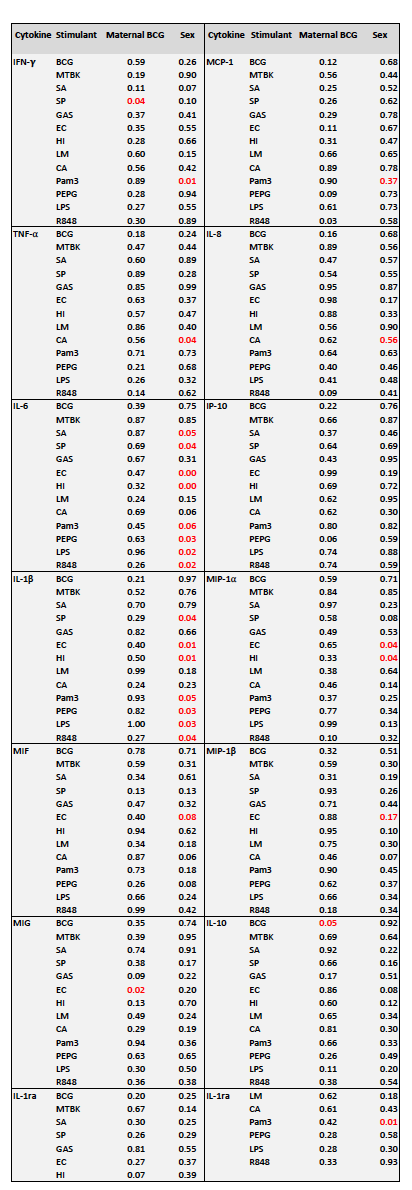


Supplementary Table 4


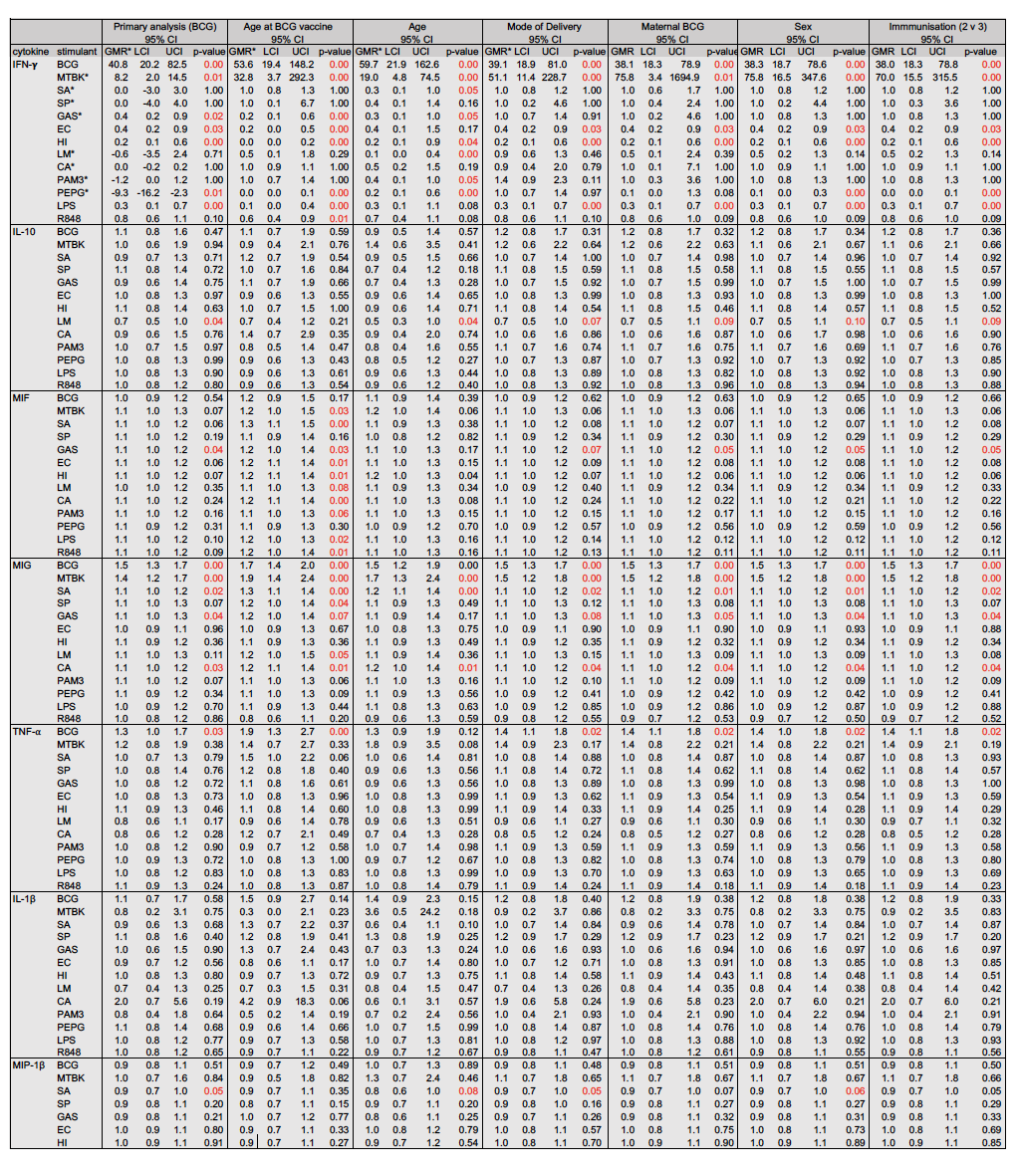
Supplementary Table 5, p1

Supplementary Table 5, p2
